# Brain M-App’s Structure and Usability: A New Application for Cognitive Rehabilitation at Home

**DOI:** 10.3389/fnhum.2022.898633

**Published:** 2022-06-17

**Authors:** Elisa Pedroli, Valentina Mancuso, Chiara Stramba-Badiale, Pietro Cipresso, Cosimo Tuena, Luca Greci, Karine Goulene, Marco Stramba-Badiale, Giuseppe Riva, Andrea Gaggioli

**Affiliations:** ^1^Applied Technology for Neuro-Psychology Lab, I.R.C.C.S. Istituto Auxologico Italiano, Milan, Italy; ^2^Faculty of Psychology, eCampus University, Novedrate, Italy; ^3^Department of Psychology, University of Turin, Turin, Italy; ^4^Department of Psychology, Universitá Cattolica del Sacro Cuore, Milan, Italy; ^5^Institute of Intelligent Industrial Technologies and Systems for Advanced Manufacturing – National Research Council, Milan, Italy; ^6^Department of Geriatrics and Cardiovascular Medicine, I.R.C.C.S. Istituto Auxologico Italiano, Milan, Italy; ^7^Humane Technology Lab, Universitá Cattolica del Sacro Cuore, Milan, Italy

**Keywords:** home-based rehabilitation, cognitive rehabilitation, virtual reality, 360° video, usability, frailty, iPad app

## Abstract

Cognitive frailty is defined as a clinical condition characterized by both physical frailty and cognitive impairment, without reaching the criteria for dementia. The major goal of rehabilitation intervention is to assist patients in performing ordinary personal duties without the assistance of another person, or at the very least to remove the need for additional support, using adaptive approaches and facilities. In this regard, home-based rehabilitation allows patients to continue an intervention begun in a hospital setting while also ensuring support and assistance when access to healthcare systems is limited, such as during the present pandemic situation. We thus present Brain m-App, a tablet-based application designed for home-based cognitive rehabilitation of frail subjects, addressing spatial memory, attention, and executive functions. This app exploits the potential of 360° videos which are well-suited to home-based rehabilitation. The Brain m-app is made up of 10 days of activities that include a variety of exercises. The activities were chosen based on those patients used to do during their clinical practice in the hospital with the aim to improve their independence and autonomy in daily tasks. The preliminary usability test, which was conducted on five older people, revealed a sufficient level of usability, however, the sample size was modest. Results from the clinical study with 10 patients, revealed that Brain m-App improved especially executive functions and memory performances.

## Introduction

The population of people aged 65 and up is expected to reach 2 billion by 2050 (Kinsella and Phillips, 2005), with major implications for health and social care planning and delivery. For instance, aging is linked to structural and physiological changes, as well as a lifelong accumulation of molecular and cellular damages caused by a complex network of maintenance and repair processes (Kirkwood, 2005). Frailty is a preclinical condition of increased vulnerability following minor stressor events, which is related with a reduction in reserve and function across a variety of physiological processes, including the ability to cope with daily or acute stresses (Fried et al., 2001; Walston et al., 2006; Eeles et al., 2012). It results from aging-related deterioration, which raises the risk of adverse effects, including falls, disability, delirium, and death (Clegg et al., 2013). Fried and colleagues (Fried et al., 2001) have operationalized this condition as the coexistence of three out of five phenotypic criteria that indicate compromised energy: low grip strength, low energy, slowed walking speed, low physical activity, and/or unintentional weight loss. However, these frailty impairments are accelerated, as are homoeostatic processes, which begin to fail at a faster rate (Ferrucci et al., 2002; Hahr, 2019).

Cognitive impairment may be linked with frailty, as Brigola et al. (2015) highlighted in their review. In fact, some recent research has begun to include cognition in the definition of frailty (Delrieu et al., 2016). Frail subjects, in particular, have deficiencies in executive, attention, free recall, and delayed free recall (Delrieu et al., 2016). Moreover, physical weakness is associated with the worst cognitive abilities (Delrieu et al., 2016). Based on these premises, “cognitive frailty” is defined as a clinical condition characterized by both physical frailty and cognitive impairment, without reaching the criteria for dementia.

Nevertheless, the state of frailty may be reversed (Gill et al., 2006; Xue, 2011) considering that many organ systems have physiological reserves needed to compensate for age-related and disease-related changes (Lipsitz, 2002).

In this regard, rehabilitation interventions attempt to restore a person’s functional capacity and avoid further deficits. The major goal is to assist patients in performing ordinary personal duties without the assistance of another person, or at the very least to remove the need for additional support, using adaptive approaches and facilities (Cameron and Kurrle, 2015). As a result, detecting frailty early and easily is critical to delaying its progression and preventing disability in the elderly. With the timely implementation of strategic rehabilitation measures, this procedure may be possible (Ma and Chan, 2020).

Depending on patient preferences and program availability, recovery services can be delivered in a wide variety of settings, including conventional hospital rehabilitation wards, outpatient facilities, acute hospital wards, and high-dependency units (Cameron and Kurrle, 2015). One example of an interdisciplinary rehabilitation program is the “Frailty Intervention Trial Program” (Fairhall et al., 2015) which includes geriatrics, physiotherapy, nursing, psychology, and health economics professionals.

Several personal health care and aging technologies have recently been proposed to help prevent, mitigate, or alleviate some of the most common illnesses that plague the aged.

New technology can be used in practically any situation where weak patients require assistance (Mugueta-Aguinaga and Garcia-Zapirain, 2017; Gallucci et al., 2021) for both motor and cognitive purposes (Pedroli et al., 2016, 2019b; Serino et al., 2017b). Among all technologies, Virtual Reality (VR) is one of the most successful examples of applications for cognitive rehabilitation (Riva et al., 2020). In this section, we will focus on its use for cognitive rehabilitation.

VR is a computer-generated technology that simulates life-like environments artificially, providing a “*convincing illusion and a sensation of being inside an artificial world that exists only in the computer*” (Tieri et al., 2018; Riva et al., 2019). Advances in virtual technologies have created platforms for displaying 3D objects in a dynamic, consistent, and accurate manner, allowing for the presentation of complex stimuli in a way that allows for both careful monitoring of laboratory measures and naturalistic observation of real-world situations (Matheis et al., 2007; Parsons, 2015).

Virtual-based interventions provide users a sense of presence or the sensation of being there in the virtual world. This sensation might be classified as a neuropsychological phenomenon resulting from our biological heritage and our experiences as active agents (Riva, 2008). The potential of virtual reality is based on the display and management of complex perceptual inputs that may be utilized to test neurocognitive and affective perception while participants are immersed in realistic simulations.

According to Riva and colleagues (Riva et al., 2006), individuals can benefit from the VR experience and boost their engagement to produce better results, by developing challenging and interactive tasks, resulting in promising cognitive and physical rehabilitation outcomes (Robinson et al., 2015; Mancuso et al., 2020; Riva et al., 2020).

This technology has been used in several therapeutic procedures. Among them, Pedroli et al. (2019a) proposed a VR-based motor protocol for frailty rehabilitation, implying a dual-task intervention (cognitive and motor).

In this regard, 360° videos are a newer technology that preserves many of the benefits of VR at a cheaper cost in terms of time and resources. They’re spherical recordings captured by sophisticated cameras with omnidirectional lenses that can collect images from all over the scene. Users can glance about the area as they would in a real-life scenario. Users can control the viewing direction by moving their head: looking up, they can see the sky/roof; looking down, they can see the floor/ground; and moving their head, they can see what is going on all around them. They’ve been used in a number of contests because of their adaptability, affordability, low prices, and ease of usage. Furthermore, 360° movies can be seen from a variety of perspectives: for example, if the camera is mounted on the user’s head while the video is being recorded, the user can experience the world from a first-person perspective. Otherwise, the user can enjoy the scene as an outside spectator by placing the camera in any section of it (third-person perspective). Thus, 360° videos can give immersive experiences by instilling a sense of presence, allowing to concentrate on the virtual experience by making users feel physically present in the scene. Several studies have shown that 360° videos are similar to real-world environments in terms of psychological, physiological (Higuera-Trujillo et al., 2017), and cortical responses (Robertson et al., 2016), despite the fact that they have not been properly regarded as realistic VR technology, providing a sub-optimal experience.

Additionally, these technologies are especially well-suited to home-based rehabilitation. Home-based rehabilitation activities can be an effective means of reducing frailty and decreasing cognitive decline. These initiatives are one of the most recent responses to the requirements of the aging and frail population, fostering independence, ensuring independent home-living, and resulting in positive quality-of-life outcomes (Stones and Gullifer, 2016). Moreover, *aging in place* is undoubtedly preferred by older people because it gives them control and influence on their lives, helping them preserve their identity and wellbeing (Cutchin, 2004). Residential solutions, on the other hand, might cause emotional stress, despair, loneliness, adaptation challenges, functional degradation, and diminished wellbeing (Chapin and Dobbs-Kepper, 2001).

Even for frailty rehabilitation, the promise of technology-based remote treatments in boosting elder independence has been well recognized (Kim et al., 2017; Serino et al., 2017a). For example, a study used 360° technologies to create a home-based motor rehabilitation program that can be accessed by watching movies on an iPad while doing cycling activities (Pedroli et al., 2020).

Overall, home-based rehabilitation allows patients to continue an intervention begun in a hospital setting while also ensuring support and assistance when access to health-care systems is limited, such as during the present pandemic situation.

Based on these premises, in the following section, we present Brain m-App, a tablet-based application designed for home-based cognitive rehabilitation of frail subjects, addressing spatial memory, attention, and executive functions. The app was created using the authors’ immersive High-end VR cognitive protocol described in a previous study (Pedroli et al., 2019a). The goal of this home-based cognitive app is to ensure that the rehabilitation process runs smoothly between the hospital and the patient’s home. Although using a Head-Mounted Display is advised for improved immersion and a sense of presence, it is not recommended for usage on its own without assistance for safety reasons. As a result, we created this tablet application to overcome this limitation, allowing 360° videos to be explored without the use of an immersive gear. The results of the first usability study are presented; the usability methodology is based on a previous paper (Pedroli et al., 2020), in which the authors designed and evaluated another home-based motor rehabilitation application. A pilot investigation of clinical use is discussed in the final section.

## Brain m-App

Brain m-App was born within a project aimed at continuing the high-end rehabilitation started in the hospital at home. The theoretical foundation of Brain m-App is described in a previous paper (Pedroli et al., 2019a). The design process was based on the User-Centered Design (UCD). In particular we simplified the structure of the tasks, we made the things visible, we planned for possible errors and generally we made it easy to determine what actions are possible at any moment. These principles allow us to consider the global experience of the subjects during the interaction with the application (Gaggioli et al., 2017).

### Description

**FIGURE 1:**
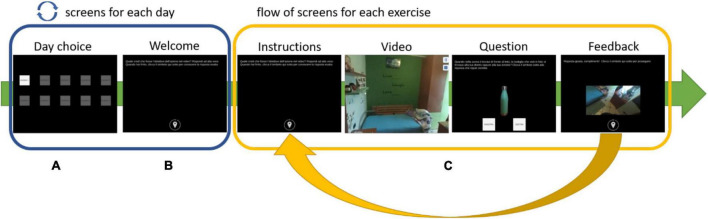
Exercise’s flow: **(A)** select the day, **(B)** read the instructions, **(C)** watch a video, answer the question, and have feedback.

The app starts with a screen that displays 10 buttons, each representing one of the training days. To make things easier, the app allows users to choose only the appropriate training day. Furthermore, the user’s options inside each screen are limited to reduce the risk of incorrect adherence to the procedure during the performance. Once the user begins the daily training session, he or she is only allowed to click on the buttons to advance to the next screen, pause or resume a video, and answer questions, with no ability to go backward. Every daily training session starts with a welcome screen that provides information about the session (number of the day) and reminds to perform each activity according to the instructions. When the user selects the button at the bottom of the welcome screen, the training begins. Depending on the training day, the user is presented with a variety of exercises ranging from 7 to 9, all of which follow the same flow (see Figure 1). The training day is complete when all of the exercises have been completed, and the application prevents it from being repeated.

### Application Architecture

**FIGURE 2:**
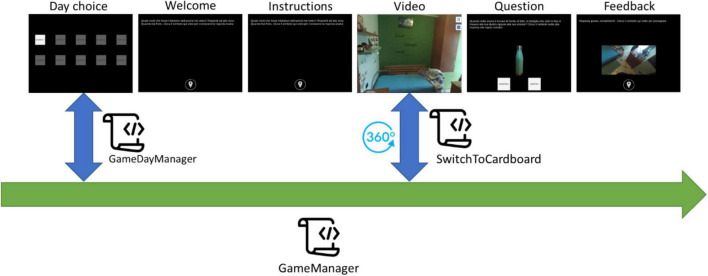
Architecture of the application.

The application has been developed using Unity, a cross-platform game engine developed by Unity Technologies. The application consists of a set of *ad hoc* scripts, C# code executed during the “play” state, managing the game’s behavior during its different tasks. Figure 2 shows the architecture of the application and the interaction between the scripts. The GameManager script is the core of the application. It is responsible for providing the graphic contents, texts, images, videos, audio, and buttons required by each step of the training session. It is in charge of managing the user’s interactions with the contents generated by giving positive or negative acoustic feedback depending on the user’s answer and showing texts describing the user’s choice results. Moreover, it manages the flow of the training session steps and, if the daily training session has been completed, it saves the information on the device, preventing the user from repeating the session. The (GM) script invokes the GameDayManager (GDM) script at each application launch. GDM is responsible for establishing if the current date is different from the one saved in memory. No date saved in the memory means that the application has never been launched before by the current user, and so the application will activate only the day 1 button. If a date is saved in memory and it is the same as the current date means that the user has already performed the training session: if the daily session has been completed, the buttons to choose the training day are all disabled, otherwise, the session is interrupted, and the user can repeat it from the beginning by clicking on the active button (current day) in the Day choice screen.

Lastly, the GM script invokes the SwitchToCardboard script whenever the video is shown as a 360° video, allowing the user to view it in multiple directions from a fixed central point using the device’s gyroscope sensor.

### Tasks

The Brain m-app is made up of 10 days of activities that include a variety of exercises. The activities were chosen based on those that patients used to do during their clinical practice in the hospital. They want to improve their independence and autonomy in daily tasks by training spatial memory, executive functions, and attention skills. Patients were asked to participate in two activities (days) every week for a total of 5 weeks, based on a previous pilot study which reported significant results after 10 sessions (Serino et al., 2017a). Every day, roughly 30 min are required. All of the tasks require patients to watch a 360° video while facing or holding the iPad. There are 22 distinct sorts of exercises, ranging in difficulty from easy to challenging, spread out across the 10 days. A group of exercises consists of watching the video and identifying, saying it aloud, the aim of the action, e.g., tidying the room or saying all the actions performed or listing the actions seen or recognizing unnecessary actions. All of these activities targeted attention and executive functions (planification, working memory, and inhibition of unnecessary information). Another group of exercises required to recall whether some objects were to their right or left, or if they were to the right or left of another object, after watching a video orto recall whether a certain sound (such as an alarm clock) came from their right or left. These exercises required patients to encode and then recall some information from both an egocentric and allocentric perspective (spatial memory). A tougher assignment requires patients to recognize the map of a home after exploring it by clicking on a sign located on each door as they move through the rooms. Another is to get to one room (for example, the bedroom) by starting from another (for example, the kitchen) and without passing through another (e.g., bathroom). Another activity requires patients to walk through the house and count how many target objects (such as tennis balls) they come across. All these exercises aimed to train both working memory and spatial memory. Patients were encouraged to appropriately set up the room before beginning a new session (e.g., establish a place without carpets, domestic animals, or canes) and to wear appropriate, comfortable shoes to protect their safety.

## Usability Test

When new tools and technology are developed, usability should be analyzed, as one of the most important aspects of human-technology interaction. Usability can be defined as the degree to which a subject can use a given system to achieve specific goals *effectively* (the possibility for the users to achieve goals), *efficiently* (the effort made by the users to reach the goal), and *satisfactorily* (what users think about the interaction with the system) within a well-defined context of use (Argent et al., 2018). A recent systematic review by Tuena and colleagues (Tuena et al., 2020) outlined that to evaluate the usability of an application for an elderly population it is necessary to:

(1) identify obstacles and facilitators
(2) develop appropriate tasks for the sample
(3) define the usability criteria
(4) test its clinical use.

In the present study, usability has been assessed using The System Usability Scale (SUS) (Brooke, 1996), the Senior technology Acceptance Model (STAM) (Chen and Chan, 2014), the Thinking Aloud protocol (TAP) (Lewis, 1982) and a part of the Independent Television Commission Sense of Presence Inventory (ITC-SOPI) about the cybersickness (Lessiter et al., 2001). The evaluations aimed at collecting information about the usability and final users’ interaction with the technology.

SUS is a “quick and easy to use” questionnaire composed of ten items (Brooke, 1996). Subjects need to express the degree of the agreement on a Likert scale which can range from 0 to 4 for each statement. The final score can range from 0, lack of usability, to 100, optimal usability. The qualitative interpretation of the score was developed by Bangor and colleagues (Bangor et al., 2009). The SUS has some advantages: it is adaptable to evaluate a wide range of technologies; it is fast and easy to use by both users and researchers; it provides scores easy to understand; it has a fairly low cost of administration (Bangor et al., 2009).

The STAM is a 13-item scale that investigates 4 components of the Senior Technologies Acceptance Model: beliefs, perception of control, anxiety related to technologies, and general health conditions (Chen and Chan, 2014).

The TAP (Lewis, 1982) is a technique that is generally administered to assess usability when a new technology is developed. Subjects are required to express their opinion regarding the technology employment and criticism while performing the task. The observer (e.g., the therapist in our study) is asked to take notes of participants’ observations and concerns without attempting to interpret their actions and words. All the verbalizations are transcribed and analyzed to develop the formal usability report.

The Independent Television Commission - Sense of Presence Inventory (ITC-SOPI) (Lessiter et al., 2001) is a 42-items self-report questionnaire that investigates several aspects of the VR experience. Participants are required to rate their degree of agreement-disagreement with a 5-point Likert scale. Specifically, it measures the Sense of Physical Space (SOPS), Engagement, Ecological Validity, and the Negative Effects of the VR experience. We administered only the 6 items that form the Negative Effects sub-scale, e.g., if they are tired or experience dizziness and cybersickness.

Finally, we developed a semi-structured interview to collect information about the use for the tablet (Have you ever used a tablet/iPad or smartphone/iPhone? Yes No; If so, how often do you use it in a week? rarely/sometimes/often) and, eventually, to deepen important aspects that emerged from the TAP.

### Participants

Five frail patients were recruited for the usability study at the I.R.C.C.S. Istituto Auxologico Italiano. Exclusion criteria were: (i) visual and auditory impairments that could affect the app’s usability; (ii) upper limb motor impairments that could affect the app’s usability; and (iii) a score lower of 24 on the Mini-Mental State Examination (MMSE) (Spinnler and Tognoni, 1987). All participants signed the informant consent form before the usability session. The study received ethical approval from the Ethical Committee of the Istituto Auxologico Italiano.

All the subjects’ demographic data and MMSE scores are reported in Table 1.

**TABLE 1:** Demographic data.

Subject	Age	Years of education	MMSE
1	74	13	27.7
2	71	17	27.7
3	76	12	28
4	70	18	26.3
5	76	5	24.7
MEAN	73.4	13.0	26.9
SD	2.79	5.15	1.39

### Protocol

The usability test lasted 40 min and included 5 phases:

1. Preliminary exchange of information with the patient regarding the aim of the study and the possible negative effects of 360° videos.
2. Informative consensus sign
3. Collection of subjects’ data (age, sex, education, MMSE) and their confidence with technology and electronic devices
4. Brain m-App demo session (Day 5)
5. Administration of usability questionnaires and semi-structured interviews.

Before starting the activity, the experimenter trained patients on how to turn on the iPad, interact with the icons and with the touch screen. Then, once ensured they have understood basic iPad operations, the device is turned off.

The experimenter then gave these directions. “Open the iPad app store and search for “Auxologico 360.” Choose “Day 5” and follow the on-screen directions. As required by the TAP, the patient was asked to comment aloud on the activities while performing tasks.

To test usability, we used Day 5 since it includes a variety of exercises that patients could encounter all over the days. The activity starts with: “Welcome to the fifth session of the training path! Follow the instructions to complete the exercises. Click the symbol below when you are ready to start.”

In Exercise 1, patients have to watch a video while paying close attention to the actions taken. Following that, it is necessary to explain aloud, listing any steps that may have preceded the one just demonstrated.

Exercise 2 demands to watch the same video a second time, this time paying close attention to the setting and the objects. The user is now asked to recall whether certain objects (such as a pasta pan) were to the right or left of others while they were in a specific position (e.g., in front of the stove). In exercise 3, the patients are given another video and asked to concentrate on the objects. Then they are asked to recall the relative positions of various items.

In exercises 4 and 6, they must watch another video while paying close attention to the actions taken. “You saw yourself acting to PREPARE THE SUITCASE in the video. While doing so, some unnecessary actions were carried out that had nothing to do with the suitcase’s preparation. Make a list of all the things you’ve done that aren’t necessary.”

Exercises 5 and 7 require moving the iPad to actively explore the house that will be shown, moving between the rooms. To change rooms, they must click on the sign above each door. The exploration of the house will come to an end when you select the “EXIT” icon on the front door. The goal is to choose the map that best describes the house they’ve just explored from those displayed on the screen.

Finally, patients were instructed to choose the Day 6 symbol and then turn off the iPad. The list of tasks that were analyzed during the TAP is presented in the first column of Table 2.

**TABLE 2:** Results of the thinking aloud protocol.

Tasks	Problem	Frequency	Solution
**Use of the tablet**			
Switch on	Difficulty to find the tablet’s “switch-on” button	1	
Switch off	None		
**Instructions**			
Listening	None	None	
Comprehension	None	None	
Application interaction	Impossibility to pause the exercises	5	To include a pause button
	Impossibility to restart each exercise	5	To insert a key to go back to the previous exercise
**Day 5 exercise 1**			
Listening	None	None	
Comprehension	Difficulty in understanding the instruction to list the actions that may have preceded the one just shown	3	To simplify the instructions
Execution	Difficulty in interacting with exercise progress buttons	1	To explicit/highlight the progress button
**Day 5 exercise 2**			
Listening	None	None	
Comprehension	Difficulty in understanding the instruction of the spatial memory exercise	1	To simplify the instructions
	The user recognizes the video, without comprehending the required activity	1	To highlight that this is a new exercise
Execution	None	None	
**Day 5 exercise 3**			
Listening	None	None	
Comprehension	Difficulty in understanding the instruction of the spatial memory exercise	2	To simplify the instructions
Execution	Difficulty in recognizing some objects because of the poor video quality	3	To improve video quality or to choose a more visible target object
	The user has been interrupted during the execution and he/she does not have the opportunity to go back to the activity	1	To insert a key to go back to the previous exercise
**Day 5 exercise 4**			
Listening	None	None	
Comprehension	None	None	
Execution	None	None	
**Day 5 exercise 5**			
Listening	None	None	
Comprehension	Difficulty in understanding/remembering the instruction to select the map that best describes the house they have just explored	2	
Execution	Difficulty in interacting with the tablet during the house exploration	2	To include a training for the exploration of 360° videos
	The user selects the exit button without exploring the house	2	To include a training for the exploration of 360° videos
	Difficulty in interacting with exercise progress buttons	3	To explicit/Highlight the progress button
	They execute the previous exercise	2	
	Difficulty in selecting the “Exit” button	2	
**Day 5 exercise 6**			
Listening	None	None	
Comprehension	None	None	
Execution	The user looks tired	2	To include a pause button
	The user lists every single activity even the useless ones	2	
	Difficulty in interacting with exercise progress buttons	1	To explicit/Highlight the progress button
**Day 5 exercise 7**			
Listening	None	None	
Comprehension	Difficulty in understanding the instruction	2	To simplify the instructions and to repeat the aim of the exercise before showing the video
Execution	Difficulty in interacting with the tablet especially during the exploration of the house	2	To include a training for the exploration of 360° videos
	Difficulty in interacting with exercise progress buttons	1	To explicit/Highlight the progress button
	They execute the previous exercise	2	
	Difficulty in finding the “Exit” button	2	
	Uncertainty of having completed the exercise	2	

## Results

**FIGURE 3:**
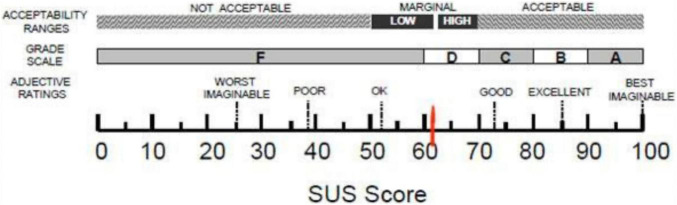
Results of the SUS scale.

Starting from quantitative data, the mean score of the SUS is 61.0 (*SD* = 22.0). According to Bangor and colleagues (Bangor et al., 2009) this score places our app in a marginal zone between High and Low acceptability and level of usability that can be defined as “ok,” as shown in Figure 3.

Analyzing the information about the use of the tablet, we notice that 2 out of 5 subjects have never used this technology and, among those who have used it, one reported using it often while the other rarely. Table 3 shows the results of the questionnaires used.

**TABLE 3:** Results of usability questionnaires.

Subject	SUS	Tablet use	Frequency	Negative effects	Stam attitude	Stam control	Stam anxiety	Stam health
1	75	0	0	1	8.3	8.75	4	8.8
2	77.5	1	1	1.3	7.7	7.75	3.5	8.2
3	55	1	1	1	4.7	7	4.5	9
4	72.5	1	3	1	10	8.75	1	8.6
5	25	0	0	1.2	6.3	6.25	2	7.4
**MEAN**	**61.0**	–	–	**1.1**	**7.4**	**7.7**	**3**	**8.4**
**SD**	**22**	–	–	**0.14**	**2.01**	**1.10**	**1.46**	**0.63**

The results of the Stam Scale results reveal that our sample has a positive attitude toward technology (*M* = 7.4/10; *SD* = 2.01), has good control/access to technological devices (*M* = 7.7/10; *SD* = 1.1), has a low level of technology-related anxiety (*M* = 3/10; *SD* = 1.46), and considers themselves in good health conditions (*M* = 8.4/10; *SD* = 0.63).

As shown by the ITC-SOPI sub-scale investigating negative effects, all subjects reported minimal side effects (*M* = 1.1/5; *SD* = 0.14).

Qualitative results of the thinking aloud protocol are shown in Table 2 is structured as follows:

1 column: description of the task
2 column: problem encountered by patients
3 column: number of patients that encountered that problem
4 column: some possible solution for that problem

Overall, patients reported more difficulties in the 5th exercise. They had to actively explore the house by moving the iPad around and between rooms until they saw the Exit button. They completed the experiment by choosing the map that best described the house they had just explored from the options displayed on the screen. Two patients either could not understand or forgot the instructions during the activity. Two patients used the “Exit” button instead of exploring the house; three persons had trouble interacting with the exercise progress buttons that allowed them to explore the house. Two participants followed the instructions from the prior activities, while other two had trouble clicking the “Exit” button. A viable answer to all of the aforementioned issues could be training in which patients move the iPad around to explore 360° settings. The exercise will be easier to understand as a result of this instruction, and the progression buttons will be more accessible. The house maps were not realistic, according to one patient, who suggested simplifying the activity by reducing the house’s size and number of rooms. Another patient had difficulty with the activity as well. To address these issues, we will allow patients to pick between two house maps rather than four. Overall, the patients had some issues interacting with the Brain M-app: they were unable to interrupt the exercises and return to the prior activities or instructions. To address this issue, we’ll include a pause button that will allow you to read the instructions again before beginning the exercise.

## Clinical Pilot

### Sample

We involve nine patients with Mild Neurocognitive Disorder (World Health Organization, 2019) for the clinical pilot study, divided into two groups. Four patients are in the experimental group (EG) (Brain M-App condition) and five in the control group (CG) (Treatment-As-Usual, TAU). The demographic data (age, years of education, and MMSE) are in Table 4.

**TABLE 4:** Demographic data.

	Group	Years	Education	MMSE
				
Mean	CG	74.2	9.40	25.6
	EG	79.5	13.0	27.9
Standard deviation	CG	3.96	3.51	2.14
	EG	0.577	4.08	0.532

The first one (EG) uses Brain M-App at home for 10 sessions three times a week, while the CG performed 10 sessions of classical paper and pencil exercises at home as well. The exercises of the CG are like those of Brain M-app and are also proposed with increasing difficulty. For example, the patient will have to memorize the matrices or images and then recall them, reorder actions or schedule workdays, observe a picture and then remember the position of some objects, describe actions aimed at reaching a specific goal. All these exercises aimed at training executive functions, attention and spatial memory.

### Methods

We used two measures of clinical change to assess the effectiveness of the VR-based (Brain M-app) and TAU intervention, namely the reliable change index (RCI) and the clinically significant change (CSC) (Evans et al., 1998). The former measure if change in the test score is due to measuring unreliability, the latter measure effective clinical change in the examined test in the patient. Normative data of the MMSE (Magni et al., 1996), Frontal Assessment Battery (FAB) (Appollonio et al., 2005), Corsi block-tapping test (Spinnler and Tognoni, 1987), Forward Digit Span (FDS; Spinnler and Tognoni, 1998), Phonemic Verbal Fluency (PVF) (Novelli et al., 1986), Semantic Verbal Fluency (SVF) (Novelli et al., 1986), story recall (Spinnler and Tognoni, 1987), and Corsi supra-span (Spinnler and Tognoni, 1987) was used. For the computation of RCI and CSC of the story recall, Corsi block-tapping test and supra-span, and FDS a measure of test-retest was missing in the normative data, and we used a hypothetical Cronbach’s alpha of 0.90. Jacobson-Truax plot was used to depict the RCI-CSC findings (Evans et al., 1998). The plot shows the RCI 95%CI and the CSC cut-off. RCI is divided into unchanged, RCI improvement, and RCI deterioration between pre-test and post-test evaluations. Results are reported in percentage, expressing the percentage of patients improved, deteriorated, and unchanged on the total, and for the CSC the percentage of patients with clinical change on the total of RCI improved.

## Results

**FIGURE 4:**
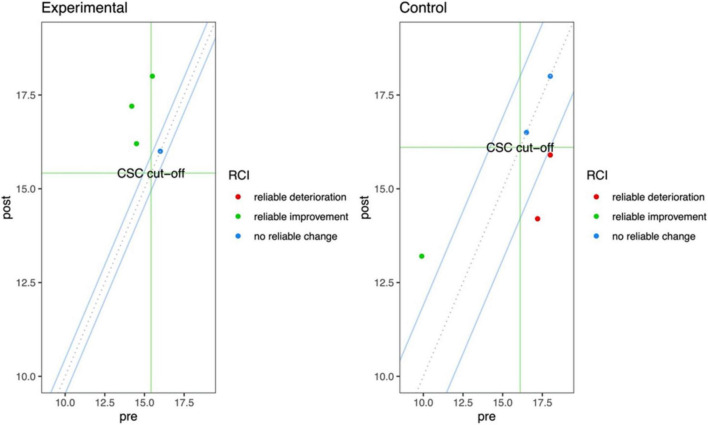
Jacobson-Truax plot shows the RCI 95%CI (blue lines) and the CSC cut-off (green lines). The upper-left rectangle delimited by the green CSC lines shows the area of significant clinical change. The upper-right and lower-left rectangles delimited by the green csc lines show they are of RCI without a CSC.

Table 5 shows the findings regarding the clinical change in the patients for each neuropsychological test. The VR training improved especially (i.e., patients with CSC) the FAB, the story recall, and the Corsi block-tapping test, whereas the TAU improved the Corsi supra-span, the Corsi block-tapping test, and the MMSE. A reliable change without CSC was found for the SVF in the VR group and the FAB for the TAU. Figure 4 shows the FAB results of the VR training compared to the TAU. The pictures highlight that two out of four patients of the experimental group show a clinical improvement beyond the measurement error (the dots in the upper left dial). On the other hand, none of the subjects in the CG showed clinical improvement; the performance of two of them can be classified as “reliable deterioration.”

**TABLE 5:** Results of VR and TAU training.

			RCI			
	
Test	Improvement (*CSC*)		Deterioration		Unchanged	
			
Group	VR	TAU	VR	TAU	VR	TAU
MMSE	25%	17% *100%*	50%	0%	0%	83%
FAB	75% *66%*	20%	0%	40%	25%	40%
Corsi	25% *100%*	40% *40%*	25%	0%	50%	60%
FDS	0%	0%	50%	0%	50%	100%
PVF	0%	0%	50%	0%	50%	100%
SVF	33%	0%	0%	0%	67%	100%
Story recall	25% *100%*	0%	0%	60%	75%	40%
Corsi supra-span	33%	60% *66%*	33%	0%	34%	40%

*Italic values represent the percentage of patients with a CSC on the total of patients with RCI improvement. Non-italic percentages represent RCIof the total of the included patients in the analyses.*

## Discussion

Aging is a process characterized by a loss of physical, sensory, and mental abilities, as well as increased morbidity and multimorbidity, which can lead to disability. Thus, the main goal of successful aging management is to keep older people healthy, active, and independent for as long as possible, addressing functional decline. Using assistive health technology (AHT; i.e., technologies devoted to maintaining or improving functionality, autonomy, and wellbeing) or medical devices (MD; i.e., technologies used for prevention, diagnosis, and treatment) may also benefit older people (Garçon et al., 2016). However, ensuring accessibility and use of these technologies among the elderly is crucial (World Health Organization, 2015; Beard et al., 2016).

In this research, an innovative tablet device for cognitive home-rehabilitation for frail people is presented. The preliminary usability test, which was conducted on five older people, revealed a sufficient level of usability, although the sample size was modest. This approach is one promising option for continuing cognitive rehabilitation that began in the hospital with the therapist and continues at home. The therapist may normally provide cognitive exercises and activities at the end of the inpatient treatment to guarantee that the rehabilitation can continue at home. Unfortunately, without a therapist or help, patients might have difficulty completing the exercises on their own. Our system is intended to serve as a guide for people undergoing cognitive-home rehabilitation. In addition, our app exploits 360° video technology which results in a higher ecological validity, a higher engagement in a simple way. For instance, it is critical to develop an easy-to-use system with a strong theoretical foundation. Although the small number of individuals participating in this study, the homogeneity of the data obtained from the usability interviews allows us to hypothesize that once the difficulties that emerged through usability study are resolved, Brain m-App might achieve a higher level of usability. In addition, other studies have conducted usability tests with small groups of participants (between 4 and 7) (Yip and Man, 2009; Kiselev et al., 2015; Pedroli et al., 2018, 2020; Riaz et al., 2021; Webber et al., 2021). Moreover, according to Virzi (1992), a sample of four or five subjects could detect 80% of the usability issues; more subjects might reveal less and less new information; and the most severe usability issues are likely to have been detected in the first few subjects.

The issues that emerged from the usability study are not related to the application’s structure and the updated version may require some small interventions. For this purpose, a new usability test will be performed after the upgrade of the application with a larger sample.

Regarding the pilot clinical trial, our sample has some critical issues like the limited size and the diversity in MMSE scores. Despite these limitations, it seems that our software may improve target clinical domains such as verbal-auditory memory and executive functioning. In fact, for the FAB and Story Recall, the benefit is greater than traditional training, and for the Corsi block-tapping test, it is comparable. From the clinical point of view, further research is needed to get more conclusive results, but the ones presented here are promising. So, the next steps will regard new clinical testing with a larger group of both frailty and Mild Neurocognitive Disorder patients. We’ll see how effective our software is comparable to traditional paper and pencil tasks. Moreover, we will develop new tasks targeting various cognitive domains like attention, verbal and semantic memory, language and visuo-spatial abilities.

## Data Availability Statement

The raw data supporting the conclusions of this article will be made available by the authors, without undue reservation.

## Ethics Statement

The studies involving human participants were reviewed and approved by the Ethical Committee of Istituto Auxologico Italiano. The patients/participants provided their written informed consent to participate in this study.

## Author Contributions

EP, VM, and PC: conceptualization. CT, VM, and PC: methodology. EP, VM, and CS-B: writing—original draft. CT, PC, VM, and EP: data curation and analyses. All authors have read and agreed to the published version of the manuscript.

## Conflict of Interest

The authors declare that the research was conducted in the absence of any commercial or financial relationships that could be construed as a potential conflict of interest.

## Publisher’s Note

All claims expressed in this article are solely those of the authors and do not necessarily represent those of their affiliated organizations, or those of the publisher, the editors and the reviewers. Any product that may be evaluated in this article, or claim that may be made by its manufacturer, is not guaranteed or endorsed by the publisher.
